# Exploring the Antihyperglycemic Chemical Composition and Mechanisms of Tea Using Molecular Docking

**DOI:** 10.1155/2020/8871088

**Published:** 2020-12-02

**Authors:** Yue Sun, Lufei Wang, Lily K. Shaughnessy, Yan Lin, Qingliang Xu, Xueping Shi, Liang Zhang, Rilei Yu, Hang Xiao, Xiaochun Wan, Xian Wu

**Affiliations:** ^1^State Key Laboratory of Tea Plant Biology and Utilization, Anhui Engineering Laboratory for Agro-Products Processing, Anhui Agricultural University, Hefei 230036, China; ^2^MOE Key Laboratory of Contemporary Anthropology, B & R International Joint Laboratory of Eurasian Anthropology, School of Life Sciences, Fudan University, Shanghai 200438, China; ^3^Department of Chemistry and Biochemistry, Miami University, Oxford, OH 45056, USA; ^4^School of Pharmacy, Ocean University of China, Qingdao 266100, China; ^5^School of Engineering and Technology, Jiangsu Institute of Commerce, Nanjing 211168, China; ^6^Department of Food Science, University of Massachusetts, Amherst, MA 01003, USA; ^7^Department of Kinesiology, Nutrition and Health, Miami University, Oxford, OH 45056, USA

## Abstract

Tea, a widely consumed beverage, has long been utilized for promoting human health with a close correlation to hyperglycemia. The Tea Metabolome Database (TMDB), the most complete and comprehensive curated collection of tea compounds data containing 1271 identified small molecule compounds from the tea plant (*Camellia sinensis*), was established previously by our research team. More recently, our studies have found that various tea types possess an antihyperglycemic effect in mice. However, the bioactive ingredients from tea have potential antihyperglycemic activity and their underlying molecular mechanisms remain unclear. In this study, we used a molecular docking approach to investigate the potential interactions between a selected 747 constituents contained in tea and 11 key protein targets of clinical antihyperglycemic drugs. According to our results, the main antihyperglycemic targets of tea composition were consistent with those of the drug rosiglitazone. The screening results showed that GCG, ECG3'Me, TMDB-01443, and CG had great target binding capacity. The results indicated that these chemicals of tea might affect hyperglycemia by acting on protein targets of rosiglitazone.

## 1. Introduction

Hyperglycemia is a common metabolic disease. The pathogenesis of hyperglycemia is complex, and it can lead to a series of serious complications. It has become a worldwide public health issue in recent decades [1]. There are many causes of hyperglycemia, such as the side effects of certain pharmaceutical drugs and hormone imbalances [2]. Diabetes is the most common form of chronic hyperglycemia that remains even in the fasted state. As of 2015, it was estimated that 415 million people in the world were suffering from diabetes, and on average, about 1.5 to 5 million people die from diabetes each year [3, 4]. Therefore, it is of great significance to develop effective, low-cost, and safe approaches to prevent, delay, or even reverse hyperglycemia. Epidemiological studies showed that consistent long-term consumption of fruits and vegetables may reduce the risk of hyperglycemia. Phytochemicals, such as polyphenols and flavonoids, are bioactive components found in fruits and vegetables that contribute to antihyperglycemic activities [5, 6]. Thus, these dietary phenolic compounds which play a potential role in alleviating hyperglycemia deserve in-depth study.

**Figure 1:**
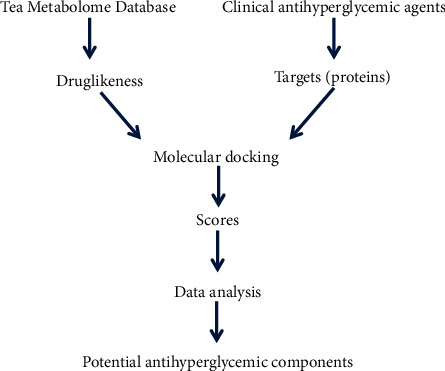
Diagrammatic scheme for exploring the antihyperglycemic components in the tea.

The Tea Metabolome Database (TMDB) has already been established by the State Key Laboratory of Tea Biology and Utilization, Anhui Agricultural University, and 1271 small molecule compounds have been identified from the tea plant (*Camellia sinensis*) to date [7]. Moreover, it has been found that tea with different processing procedures produced antihyperglycemic functions *in vivo* and *in vitro*. Han et al. made high-fat diet mice free to drink tea infusion and compared the antihyperglycemic efficacy of green tea, black tea, and yellow tea. The results showed that while they all had a certain degree of antihyperglycemic activity, yellow tea showed the strongest efficacy in this model [8]. Xu et al. found that yellow tea, green tea, black tea, and white tea had a lipid-lowering effect at the cellular level. They further studied the mechanisms of yellow tea *in vivo* and found that yellow tea not only inhibited the production of TNF-*α*, MCP-1, IFN-*γ*, IL-6, IL-1*β*, and other serum inflammatory cytokines but also reduced blood glucose, total cholesterol (TC), triacylglycerol (TG), and low-density lipoprotein cholesterol (LDL-C) and improved glucose tolerance and insulin resistance [9]. Various pharmaceutical agents are used to treat hyperglycemia clinically, and these drugs have different physiological mechanisms that act on distinct molecular targets [10]. However, since the specific antihyperglycemic constituents and molecular targets for the tea are still unclear, the optimization of tea processing techniques and its applications as functional treatment are limited. Molecular docking is an emerging technique that predicts the ligand conformations adopted within the binding sites of molecular targets at an atomic level using computer algorithms. If the structures of the receptor (protein) and ligand (small molecule compound) are well defined, the chemometric method can simulate interactions between them. By predicting the structure and stability of the protein-ligand complex, the potential bioactivity of the ligand is evaluated [11]. Due to the fast screening and low cost of the molecular docking method, the technology has developed rapidly in recent years. It has been widely used in the design of drug molecules and the screening of bioactive ingredients in traditional Chinese medicine [12, 13]. Based on the TMDB and the protein targets of clinical hypoglycemic agents, the diagrammatic scheme is shown in Figure 1. The purpose of this study was to screen out bioactive tea compounds that have potential protective effects against hyperglycemia using molecular docking technology.

## 2. Materials and Methods

The data used to support the findings of this study are included within the article or available from the corresponding author upon request.

### 2.1. Ligand Preparation and Druglikeness Analysis

1271 compounds were retrieved from the *Camellia sinensis* in TMDB and predicted for druglikeness according to Lipinski's rule of five (RO5) and the Chemistry Database (http://www.organchem.csdb.cn). The structures of all compounds were sketched in ChemDraw 8.0 and minimized in MOE, then stored in .pdf format for further molecular docking.

### 2.2. Target Protein Selection

Clinical hypoglycemic drugs were divided into five categories (Table 1). The targets of selected drugs in each category were found in DrugBank (https://www.drugbank.ca). Then the 3D crystal structures of the protein-ligand complex were found and downloaded from the Protein Data Bank (PDB; http://www.rcsb.org/pdb/home/home.do). Water molecules in the crystal structure were removed. The active site of the crystal structure was determined with the ligand.

### 2.3. Molecular Docking

The molecular docking study was performed using MOE (US Tripos 2015) and Cytoscape 3.6.0. The software operating environment was Microsoft Windows XP Professional operating system. The binding site was determined based on the location of the ligand in the crystal structure of the protein target. Five conformations were generated after each docking. The best scoring conformation with minimal binding energy was selected for further analysis. Each compound was docked with 11 protein targets. The free energy *S* value was used as the evaluation index. Since the *S* value was negative, the higher the absolute *S* value, the more stable binding activity between the ligand and protein target. A 6 to 7 absolute S value indicates a good binding force between compound and protein target, and 7+ indicates a strong binding force [14].

### 2.4. Construction of Compound-Protein Interaction Network

A compound-protein interaction network model was constructed by Cytoscape. The network included two types of nodes: compounds and protein targets. The red line indicates a strong binding force (|*S*| ≥ 7), while the black line means a good binding force (7 ≥ |*S*| ≥ 6). The network analysis revealed potential hypoglycemic components in the tea and their major targets.

## 3. Results and Discussion

### 3.1. Druglikeness Analysis

RO5 is a common rule for drug design and screening [15]. It describes the compatibility between drugs and the human body, and the oral drugs should comply with the following rules: a molecular mass less than 500 daltons, an octanol-water partition coefficient, Clog *P* not greater than 5, the number of hydrogen bond donors no more than 5, and the number of hydrogen bond acceptors no more than 10. If enough rules are violated, the drug's solubility and intestinal absorption capacity are limited [15]. With the relevant data from the aforementioned database, we used this rule to conduct initial screening. Of the 1271 tea compounds, 747 compounds complied with RO5 and had potential druglikeness.

### 3.2. Molecular Docking

**Figure 2:**
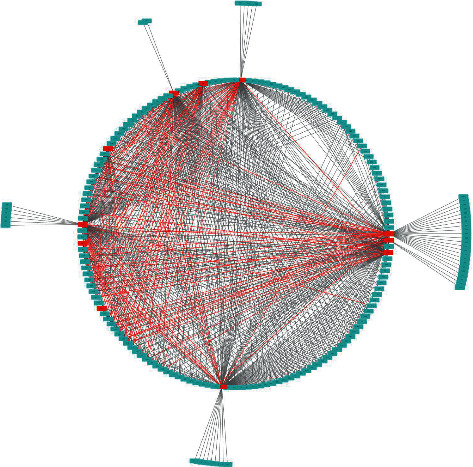
The compound-protein interaction network. Red nodes indicate protein targets and blue nodes indicate tea compounds. The red line represents a strong binding force (|*S*| ≥ 7), while the black line represents a good binding force (7 ≥ |*S*| ≥ 6).

The compound-protein interaction network model (Figure 2) includes a total of 200 nodes (11 protein targets and 189 compounds). The red line indicates a strong binding force (|*S*| ≥ 7), while the black line represents a good binding force (7 ≥ |*S*| ≥ 6). The network model revealed the complex relationship between the components of the tea and its targets by molecular docking. As shown in Table 2, the network analyzer calculated the important parameters of the network model. Crucial node degrees for the protein targets (Table 3) and tea compounds (Table 4) were demonstrated, respectively. The node degree refers to the number of lines linked to the node. The greater the degree, the more important the node in the network [16].

#### 3.2.1. Protein Target Analysis

As shown in Table 3, from the perspective of the protein targets, it was found that 11 protein targets and 189 compounds had a certain degree of binding activity, among which the best binding protein target was 5ji0, which showed a good binding force with 120 compounds (|*S*| ≥ 6). The protein target with the lowest binding degree was 5kzx, which had a good binding force with only 13 compounds. In addition, it was revealed that the degrees of binding activity of 4 protein targets (i.e., 5ji0, 2xyw, 5kjy, and 5ezv) were more than 100. 5ji0, 2xyw, and 5kjy were protein targets of thiazolidinediones (i.e., rosiglitazone). 5ezv was a protein target of biguanides (i.e., metformin). It could be speculated that the molecular targets of the hypoglycemic agent rosiglitazone might also be the main potential targets of tea, followed by metformin. On the contrary, the protein targets with poor binding degrees to the tea compounds were 1b2y, 6baa, 3lpp, and 5kzx, with degrees all less than 50. Among them, 1b2y, 3lpp, and 5kzx were targets of the hypoglycemic agent acarbose. 6baa was the target of glimepiride. Similarly, it was speculated that the effect of tea on the targets of acarbose was relatively insignificant.

#### 3.2.2. Tea Compound Analysis

As shown in Table 4, the network model was analyzed from the perspective of the tea compounds. Table 4 lists the tea compounds which had a good binding force to more than 7 protein targets. The top 10 tea compounds of each protein target were collected individually, and common tea compounds with a good binding activity to every protein target were analyzed (Table 5). Among them, GCG, ECG3'Me, and TMDB-01443 exerted the best binding affinity with 5 protein targets. CG showed good binding potential with 4 protein targets.

#### 3.2.3. Docking Interactions

**Figure 3:**
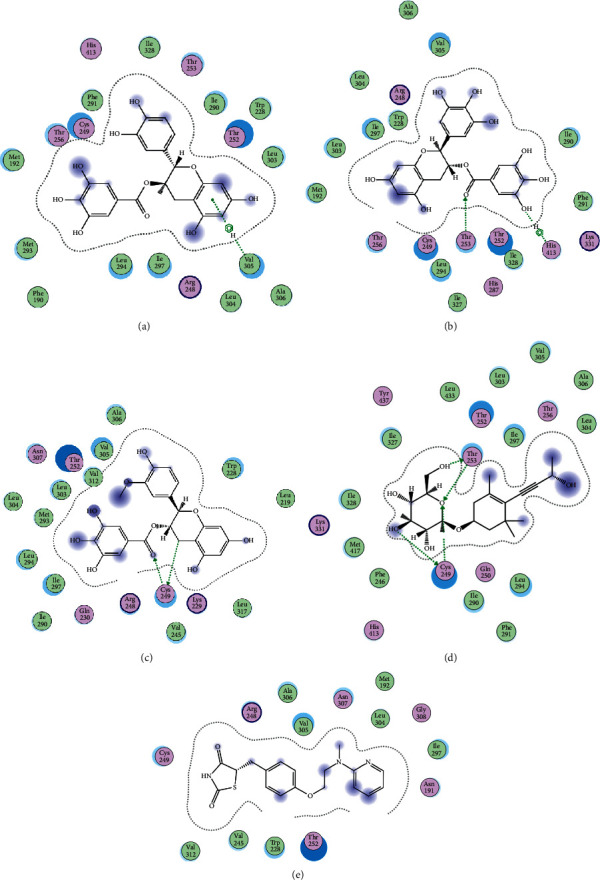
2D interactions between CG (a), GCG (b), ECG3'Me (c), TMDB-01443 (d), and rosiglitazone (e) and 2xyw by MOE-Dock.

In order to elucidate the docking pattern of tea compounds, the 2D docking interactions of clinical drugs and tea compounds with corresponding protein targets were compared. 2D interactions of CG (a), GCG (b), ECG3'Me (c), TMDB-01443 (d), and the positive control rosiglitazone (e) with 2xyw are shown in Figure 3. It was found that all 5 compounds bound to the same active pocket of protein 2xyw, and the amino acid sequence was very similar: Thr252, Ile297, Ala306, and Leu304 had a strong hydrophobic interaction with 5 compounds; Val305 showed a strong hydrophobic interaction with rosiglitazone, GCG, ECG3'Me, and TMDB-01443 and also formed a *π* − *H* interaction with CG; Trp228 and Arg248 had a hydrophobic interaction with rosiglitazone, CG, GCG, and ECG3'Me; Met192 showed hydrophobic interaction with rosiglitazone, CG, and GCG; Cys249 had hydrophobic interaction with rosiglitazone, CG, and GCG and also formed a hydrogen bond with TMDB-01443 and ECG3'Me. Cys249 contains a sulfhydryl group which could provide and receive protons to form stable hydrogen bonds.

**Figure 4:**
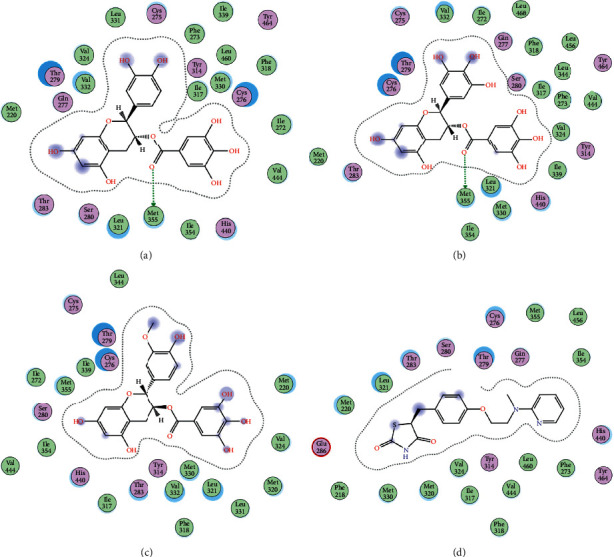
2D interactions between CG (a), GCG (b), ECG3'Me (c), and rosiglitazone (d) and 3kdt by MOE-Dock.

**Figure 5:**
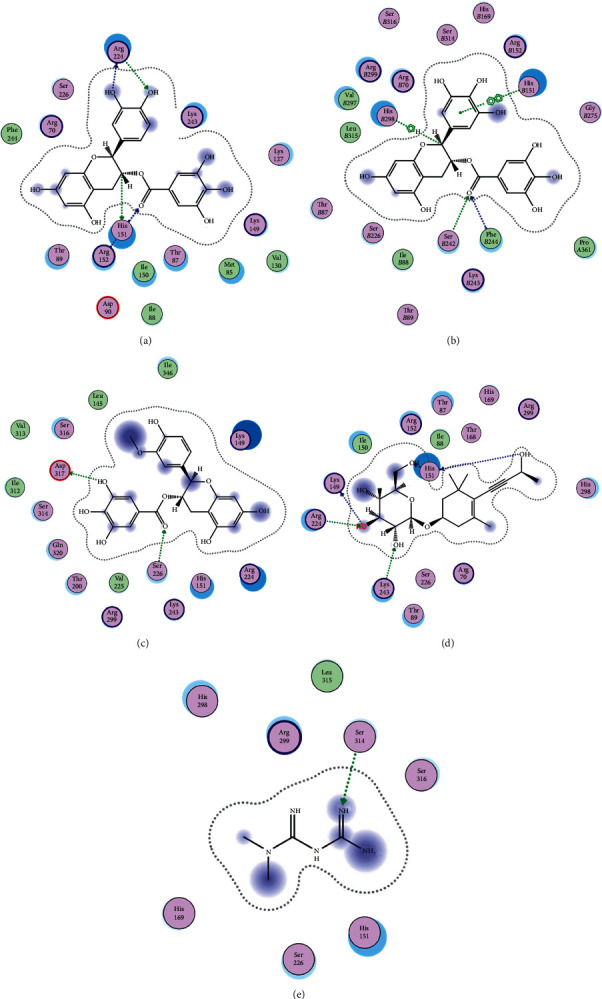
2D interactions between CG (a), GCG (b), ECG3'Me (c), TMDB-01443 (d), and metformin (e) and 5ezv by MOE-Dock.

2D interactions of CG (a), GCG (b), ECG3'Me (c), and the positive control rosiglitazone (d) with protein 3kdt are shown in Figure 4. It was found that the interactions of all four compounds bound to amino acids of 3kdt were very similar: Thr279, Cys276, His440, Leu321, Thr283, Met220, Ser280, Met330, Ile354, Val444, Phe318, Tyr314, Val324, and Ile317 had a strong hydrophobic interaction with all 4 compounds; Gln277 and Tyr464 had a strong hydrophobic interaction with CG and GCG; Met355 had a strong hydrophobic interaction with rosiglitazone and ECG3'Me. Met355 also formed hydrogen bond with CG and GCG, which might be because the methylthio group was more accessible near carbonyl bonds; 2D interactions of CG (a), GCG (b), ECG3'Me (c), TMDB-01443 (d), and the positive control metformin (e) with protein 5ezv are shown in Figure 5. It was found that the interactions of all five compounds bound to the amino acid sequence had a certain similarity: Arg299 had a strong hydrophobic interaction with metformin, ECG3'Me, and TMDB-01443; Ser316 had a strong hydrophobic interaction with metformin, GCG, and ECG3'Me; Ser314 formed a hydrogen bond with metformin and had a strong hydrophobic interaction with ECG3'Me; His169 showed a hydrophobic interaction with metformin and TMDB-01443; Leu315 had a hydrophobic interaction with metformin and GCG; His151 had a strong hydrophobic interaction with metformin and ECG3'Me and formed a hydrogen bond with CG and TMDB-01443 and showed *π* − *π* action with GCG. His151 contains an imidazolyl group which could provide and receive protons to form stable hydrogen bonds when near the alcoholic hydroxyl group, and the conjugated structure of His151 allows for the *π* − *π* effect. Ser226 had strong hydrophobic interactions with metformin, CG, GCG, and TMDB-01443 and formed a hydrogen bond with ECG3'Me. Ser226 contains an alcoholic hydroxyl group that could supply a proton to the carbonyl group of the lactone, allowing the formation of a stable hydrogen bond. His298 had a strong hydrophobic interaction with metformin and TMDB-01443 and showed *π* − *H* interaction with GCG; taken together, the interaction of tea compounds and pharmaceutical drugs with corresponding protein targets showed a certain regularity. The hydrophobic action and the hydrogen bonds of the key amino acid residues played a great role in reproducing the binding characteristics of the positive control drugs and the protein active sites.

### 3.3. Tea Compounds and the Main Protein Targets

The protein targets of thiazolidinediones (i.e., rosiglitazone) included PPAR-*α* (3kdt), PPAR-(*β*/*δ*) (2xyw), PPAR-*γ* (5u5l), RAR-RXR-*α* (5ji0), RAR-RXR-*β* (5kjy), and RAR-RXR-*γ* (2gl8). PPARs are a set of nuclear receptor proteins, and they affect lipid metabolism and adipogenesis by regulating the expression of NF-*κ*B, inflammatory and adipogenic genes. Their receptor agonists (i.e., rosiglitazone) have been used to treat hyperlipidemia and type 2 diabetes [17]. There are three subtypes of PPAR—*α*, *β*, and *γ*—which have a high degree of homology but differ significantly in tissue distribution, ligand specificity, and physiological roles. PPAR-*α* is highly expressed in the liver, intestine, and heart. PPAR-*γ* has two forms: PPAR-*γ*1 is expressed in immune cells, gut, brain, and vascular cells, and PPAR-*γ*2 is almost exclusively expressed in adipose tissues. PPAR-(*β*/*δ*) expression is very high in the small intestine and keratinocytes and also high in the liver, colon, kidney, and skin [17], in which PPAR-(*β*/*δ*) plays a critical role in regulating lipid metabolism and insulin sensitivity. Activation of PPAR-(*β*/*δ*) in adipose tissue induces expression of genes required for fatty acid oxidation and energy dissipation, which improves lipid profiles and reduces adiposity in mice [18]. According to the docking results (Table 3), PPARs (2xyw, 3kdt, and 5u5l) had higher node degrees, suggesting they were important protein targets for the tea. Previous in vivo experiments also confirmed the docking results: Yan et al. reported that green tea catechins (GTCs) could exert an antiobesity mechanism in part by modulating PPAR signaling pathways in rats. GTC supplementation significantly upregulated the levels of PPAR-(*β*/*δ*) in white adipose tissue and brown adipose tissue and increased the expression of genes involved in fatty acid oxidation in brown adipose tissue [19]. Yang et al. added green tea polyphenols (GTPs) in the drinking water of rats and found that the GTP treatment upregulated SIRT3 and PPAR-*α* expression, increasing the PPAR-*α* mRNA level [20].

Retinoic acid receptors (RARs) and retinol X receptors (RXR) are two members of nuclear receptor protein families which are responsible for the transduction of retinoid signals. They could form a RAR/RXR heterodimer with three subtypes (*α*, *β*, and *γ*) [21]. The development of metabolic diseases, such as obesity and type 2 diabetes, is often associated with profound changes in the expressions of RAR/RXR involved in glucose and lipid metabolism in metabolically active cells [22]. We found that RAR/RXR-*α* (5ji0) and RAR/RXR-*β* (5kjy) had extensive binding activity with tea compounds (Table 3). This finding had been supported by many previous animal studies [23, 24]. For example, Volate et al. explored the intervention mechanism of a low oral dose of green tea infusion in mice with colorectal cancer. They showed that the expression of RXR-*α* was decreased in the positive control group, which had an early occurrence of colorectal cancer. The experimental group treated with green tea significantly increased both the protein and mRNA expression levels of RXR-*α*, suggesting that green tea infusion could inhibit the occurrence of colorectal cancer by regulating RXR-*α* [23]. Xiao et al. examined the efficacy of polyphenon E (PPE, consisting 65% of EGCG and 22% of other catechins) as a preventive agent in an azoxymethane- (AOM-) induced rat colon cancer model. They found that the expression of RXR-*α* was decreased in aberrant crypt foci (ACF) with high-grade dysplasia, and it was further decreased in adenomas and adenocarcinomas, whereas the PPE treatment partially prevented the loss of RXR-*α* expression in high-grade dysplastic ACF [24]. These results were consistent with those of the compound-protein interaction network, suggesting that our method of predicting the protein targets of tea compounds by molecular docking was reliable.

## 4. Conclusions

In this study, the molecular docking technique was applied to the virtual screening of bioactive substances in tea that has the potential to improve hyperglycemic symptoms. The bioactive components and potential targets of tea in the treatment of hyperglycemia were predicted based on the compound-target network. The main targets of tea compounds were partially the same as the drug rosiglitazone. Several components including ECG3'Me, CG, GCG, and TMDB-01443 were screened out as potential hyperglycemia improving compounds. Our study also suggested that the molecular docking technique could be used as a novel approach for studying multiaction targets of functional components of tea and other plant-based foods in the future. Further research is warranted to verify the biological efficacy of these tea compounds on their protein targets in cell culture and/or animal studies.

## Figures and Tables

**Table 1:** Clinical hypoglycemic drugs and their protein targets.

Classification	Drug	Protein target	PDB ID
Sulfonylureas	Glimepiride	ATP-sensitive inward rectifier potassium channel 11	6baa
		ATP-binding cassette subfamily C member 8	6baa

Biguanides	Metformin	5'-AMP-activated protein kinase subunit beta-1	5ezv

*α*-Glucosidase inhibitor	Acarbose	Maltase-glucoamylase, intestinal	3top
		Lysosomal alpha-glucosidase	5kzx
		Sucrase-isomaltase, intestinal	3lpp
		Pancreatic alpha-amylase	1b2y

Thiazolidinediones	Rosiglitazone	Peroxisome proliferator-activated receptor-gamma (PPAR-*γ*)	5u5l
		Peroxisome proliferator-activated receptor-alpha (PPAR-*α*)	3kdt
		Peroxisome proliferator-activated receptor-delta (PPAR-(*β*/*δ*))	2xyw
		Retinoic acid receptor RXR-alpha (RAR/RXR-*α*)	5ji0
		Retinoic acid receptor RXR-beta (RAR/RXR-*β*)	5kjy
		Retinoic acid receptor RXR-gamma (RAR/RXR-*γ*)	2gl8

Meglitinides	Repaglinide	ATP-binding cassette subfamily C member 8	6baa
		Peroxisome proliferator-activated receptor-gamma	5u5l

**Table 2:** The features of the compound-protein interaction network.

Network feature	Value
Number of nodes	200
Number of edges	740
Network density	0.037
Network heterogeneity	2.351
Average number of neighbors	7.4
Characteristic path length	2.370
Shortest paths	39800 (100%)
Network centralization	0.572

**Table 3:** The degrees of protein targets in the compound-protein interaction network.

PDB ID	Degree
5ji0	120
2xyw	116
5kjy	112
5ezv	109
3kdt	82
3top	54
5u5l	53
1b2y	35
6baa	28
3lpp	18
5kzx	13

**Table 4:** The degrees of main tea compounds in the compound-protein interaction network.

TMDB-ID	Structure	Degree
TMDB-01443	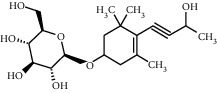	11
TMDB-00036	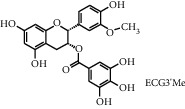	10
TMDB-00682	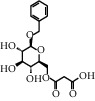	10
TMDB-null	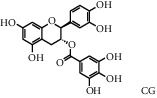	10
TMDB-00676	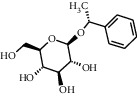	10
TMDB-00552	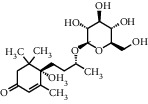	9
TMDB-01361	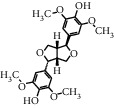	9
TMDB-01442	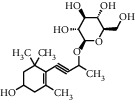	9
TMDB-00261	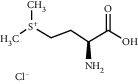	9
TMDB-01360	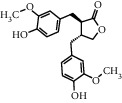	9
TMDB-00675	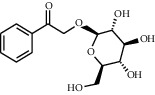	9
TMDB-00381	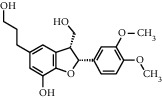	9
TMDB-01333	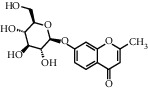	9
TMDB-00382	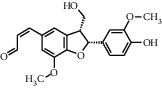	9
TMDB-00677	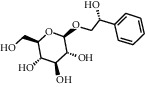	9
TMDB-00666	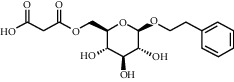	8
TMDB-00017	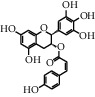	8
TMDB-00001	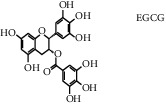	8
TMDB-00172	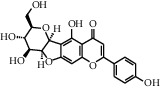	8
TMDB-null	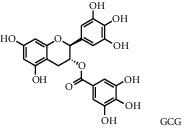	8
TMDB-00942	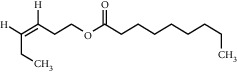	8
TMDB-00231	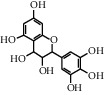	8
TMDB-00379	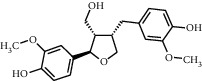	8
TMDB-01444	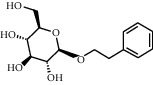	8
TMDB-00380	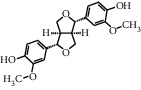	8
TMDB-01002	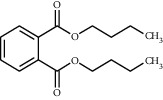	8
TMDB-01314	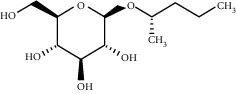	8
TMDB-00579	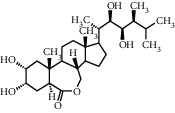	7
TMDB-00254	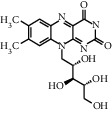	7
TMDB-01445	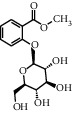	7
TMDB-01016	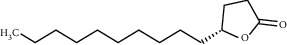	7
TMDB-00006	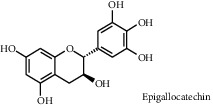	7
TMDB-00673	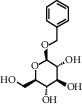	7

**Table 5:** Distribution of the top ten compounds with a good binding activity to each protein target.

TMDB-ID	Structure	Protein				
01360	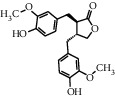	5jio	2xyw	—	—	—

01361	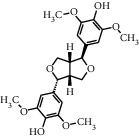	2xyw	3kdt	3top	—	—

00017	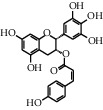	5jio	2xyw	—	—	—

Null	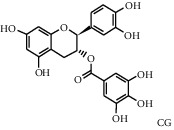	2xyw	5ezv	3kdt	6baa	—

Null	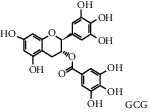	2xyw	5ezv	3kdt	6baa	5kzx

00379	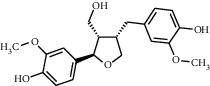	5jio	5ezv	3kdt	—	—

00036	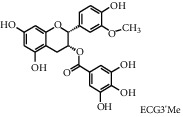	2xyw	3kdt	5u5l	5kzx	3lpp

00254	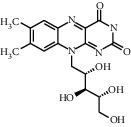	2xyw	1b2y	—	—	—

01443	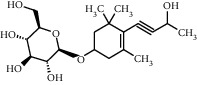	5ezv	3top	2xyw	1b2y	5jio

01442	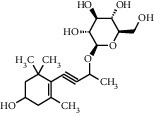	5ezv	3top	5jio	—	—

00579	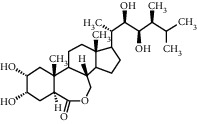	5u5l	1b2y	6baa	—	—

00381	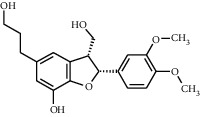	5u5l	5kzx	—	—	—

00172	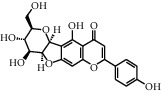	5jio	3top	—	—	—

00666	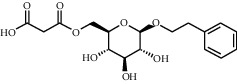	5ezv	1b2y	—	—	—

## Data Availability

The data used to support the findings of this study are included within the article or available from the corresponding author upon request.
